# Effects of medical ozone upon healthy equine joints: Clinical and laboratorial aspects

**DOI:** 10.1371/journal.pone.0197736

**Published:** 2018-05-29

**Authors:** Cynthia do Prado Vendruscolo, Juliana Junqueira Moreira, Sarah Raphaela Torquato Seidel, Joice Fülber, Henrique Macedo Neuenschwander, Giancarlo Bonagura, Fernanda Rodrigues Agreste, Raquel Yvonne Arantes Baccarin

**Affiliations:** 1 Department of Clinical Medicine, School of Veterinary Medicine and Animals Science, University of São Paulo, São Paulo, São Paulo, Brazil; 2 Department of Large Animals Clinics, Anhembi Morumbi University, São Paulo, São Paulo, Brazil; University of Umeå, SWEDEN

## Abstract

**Objective:**

The aim of this study was to verify whether transient inflammatory reactions induced by intra-articular medicinal ozone administration affect joint components, by *in vivo* evaluation of inflammatory (prostaglandin E_2_, Substance P, Interleukin-6, Interleukine-1, Tumor Necrosis Factor), anti-inflammatory (Interleukin-10) and oxidative (superoxide dismutase activity and oxidative burst) biomarkers and extracellular matrix degradation products (chondroitin sulphate and hyaluronic acid) in synovial fluid.

**Methods:**

The effects of medicinal ozone were analyzed at two ozone concentrations (groups A and B, 20 and 40 μg/ml, respectively), using oxygen-injected joints as controls (group C); each group received ten treatments (15 ml gas per treatment). Physical evaluation, evaluation of lameness, ultrasonography, and synovial fluid analysis were performed.

**Results:**

All joints presented mild and transient effusion throughout the study. Group B exhibited the highest lameness score on day 14 (P<0.05), detected by the lameness measurement system, probably because of the higher ozone concentration. All groups exhibited increased ultrasonography scores on day 14 (P < 0.05). Groups A and B exhibited increased proteins concentrations on day 21 (P<0.05). There was no change in hyaluronic acid concentration or the percentage of high-molecular weight hyaluronic acid throughout the experiment. Chondroitin sulfate concentrations decreased in group B, and did not change in group A and C, indicating that neither treatment provoked extracellular matrix catabolism. Cytokine and eicosanoid concentrations were not significantly changed.

**Conclusions:**

The ozonetherapy did not cause significant inflammation process or cartilage degradation, therefore, ozonetherapy is safe at both evaluated doses.

## Introduction

Osteoarthritis (OA) is the most common arthritic condition in humans and horses. It is the most frequent cause of lameness, leading to loss of training days and early retirement of athletes [1]. Consistent use of animals in different sports leads to a series of articular lesions, which is responsible for lameness in approximately 60% of cases [2]. OA is considered a progressive disease, affecting all articular tissues. It presents as cartilage degeneration, accompanied by mild to moderate synovial inflammation and changes in subchondral bone structure [3].

Conventional therapy usually evolving the use of non-steroidal anti-inflammatory drugs and intra-articular (IA) steroids has the objective of reduce pain and discomfort. However, because of the chronic nature of the disease, therapy is often prolonged and commonly associated with the adverse effects of anti-inflammatory drugs [4].

It has been known since 1955 [5], that a period of rest is necessary after IA steroid treatment, to allow the lesions to heal. This necessity is usually ignored, because of which “an endless destructive cycle is set in motion which, if continued, will produce a steroid arthropathy which can render the horse useless”, as written by anonymous author. Such horses usually suffer from loss of performance and are constantly medicated, which ultimately ends their athletic career.

In the search for new therapies for slowing the progression of cartilage degeneration and arthropathy and decreasing inflammation, some studies have investigated ozonetherapy and found that it can produce minimal collateral effects when correctly applied [6,7].

Ozonetherapy involves administration of medical ozone (O_3_), a mixture of oxygen (O_2_) and O_3_, composed of not less than 95% O_2_ and no more than 5% O_3_ [7]. Although medical ozone is toxic at high concentrations, it has a “therapeutic window” of concentrations ranging from 10 to 80μg/ml, where it can produce immunomodulatory, anti-inflammatory, bactericidal, anti-viral, antifungal and analgesic effects, among others [8]. At concentrations bellow 10μg/ml, O_3_ is immediately neutralized by the antioxidants in blood and is, therefore, biologically inefficient, since it does not reach the therapeutic threshold [9]. Stimulation and activation of the antioxidant system have been achieved though small and repeated oxidative shocks [6].

The objective of ozonetherapy is to cause an adequate, transitory, controlled, acute oxidative stress, not placebo effect, without leading to chronic oxidative stress. To avoid toxicity, it is extremely important to not exceed the antioxidative capacity of the organism [6].

Ozonetherapy mediates its effects through reactive oxygen species (ROS) and lipoperoxidation oxidative products (LOP), which act probably in two phases in synovial fluid (SF). The first phase involves inhibition of inflammation and reduction of prostaglandin E_2_ (PGE_2_) and pro-inflammatory cytokines concentrations through depletion of phospholipase A_2_, cyclooxygenases I and II, kallikreins, and bradykinin. Ozonetherapy could also cause a decrease in the release of serotonin and metalloproteinases, such as collagenase, aggrecanase, and gelatinase, thus avoiding the destruction of articular cartilage [6,10]. In the second phase, O_3_ mediates an increase in the concentrations of antioxidant enzymes, oxidative shock proteins (such as hemo-oxigenase-1), inhibitory cytokines (such as interleukine [IL]-4 and IL-10), and growth factors (transforming growth factor-β), it also stimulates neoangiogenesis, nitric oxide synthesis, and the release of endorphins, adrenocorticotropic hormone and cortisol. This panorama of activities mediates the articular repair process through stimulation of chondrocytes, fibroblasts, and stem cells that synthesize proteoglycans, glycosaminoglycan and collagen [6].

Because of its various effects on the body, ozonetherapy has been evaluated for efficacy and applied in treatment of several disorders, including osteomyelitis [11], chronic obstructive pulmonary disease [12], hepatitis [13], cystitis [14,15], rheumatoid arthritis [16,17], OA [18–25], back pain [26–28], multiple sclerosis [29], peritonitis [30], osseous defects [31,32], and coronary arterial disease [33]. Intra-articular ozonetherapy has been successfully used in human medicine for treatment of inflammatory diseases, chronic and acute, synovitis, capsulitis, and OA [16–25].

There are few articles on the use of intra-articular ozone in treatment of joint disease in humans, most of them are clinical reports [18,21–23,25] and one is a placebo controlled clinica trial [34], which has led to the development of some treatment protocols (Table 1) [35]. However, there are no studies involving IA O_3_ administration in horses in current literature.

**Table 1:** Intra-articular injection—treatment concepts [35].

Indication	Application form	Ozone concentration	Volume	Ozone quantity	Frequency
Arthroses	Intra-articular	7–20 μg/ml	1–20 ml	7–400 μg	1–2 x per week
	Peri-articular	2–11 μg/ml	2–5 ml	4–55 μg	1–2 x per week
FTP joint	Intra-articular	10–20 μg/ml	5–20 ml	50–400 μg	1–2 x per week
Shoulder joint	Intra-articular	10–20 μg/ml	5–20 ml	50–400 μg	1–2 x per week
Phalange joint	Intra-articular	10–20 μg/ml	1–2 ml	10–40 μg	1–2 x per week

O_3_ has emerged as a safe and feasible treatment option for various diseases in humans. Although widespread, the use of ozonetherapy suffers from lack of knowledge regarding its mechanisms of action as well as the ideal methods of administration in horses.

These shortcomings, coupled with the dearth of studies on the anti-inflammatory and antioxidant effects of medical ozone administration in equine joints, inspired this study.

## Objective

The aim of this study was to verify whether transient inflammatory reactions inducted by IA O_3_ administration affected joint components, by means of physical examination, ultrasonography, and *in vivo* evaluation of inflammatory and oxidant biomarkers and extracellular matrix degradation products in SF.

## Materials and methods

The experimental protocol used in this study was approved by the Animal Ethics Committee of the School of Veterinary Medicine and Animal Science, University of São Paulo (protocol number 6409110215; date of approval, 12/09/2015) and was carried out in accordance with the U.K. animals (Scientific Procedures) Act, 1986 and associated guidelines, EU Directive 2010/63/EU for animal experiments, https://www.gov.uk/guidance/research-and-testing-using-animals.

The study was performed at research laboratory of the Department of Clinical Medicine and at the Laboratory of Pharmacology and Toxicology, Department of Pathology, School of Veterinary Medicine and Animal Science, University of São Paulo.

### Experimental design

The horses included in the experiment were from USP research herd. They were housed in single 12 m^2^ boxes (3 x 4 m) and fed pellets (1% of the animal body weight), coast cross hay, mineral salt and water *ad libitum*. After the experiment period the animals returned to the pasture.

This study included 14 clinically healthy Arabian horses (2–4 years of age) which were non-athletes, gelded, and has no history of articular problems, thus a total of 28 tibiotarsal joints were evaluated. All horses were evaluated for lameness and subjected to clinical, radiologic and ultrasonography examination, resulting in exclusion of four joints.

The 24 selected tibiotarsal joints were randomly divided in three groups, in a way that the same horse did not received the same treatment: groups A and B (treatment groups), which received three weekly applications of 15 ml O_3_ at concentrations of 20and 40μg/ml, respectively, and group C (control group), which received three weekly applications of 15ml 100% O_2_. Each joint received a total of ten treatments. Each joint was treated only once, and the horses were allowed a 15-day period of rest between treatment of each tibiotarsal joint. The animals were assessed three times a day to any sign of discomfort.

Medical ozone was produced just before administration, using an Ozone & Life^®^ (São José dos Campos, SP, Brazil) generator, equipped with a Millex GS^®^ (Millipore, Carrigtwahill, Co. Cork, Ireland) filter; O_3_ was produced in stipulated doses by regulating O_2_ flow and electric discharge; it was packed in a Terumo^®^ (São Paulo, SP, Brazil) latex-free syringe and held in vertical position to prevent gas leakage until use. Oxygen syringes were filled just before use, directly from an oxygen cylinder, using a 0.22μm filter.

### Sample size calculation

The sample size calculation was determined to provide an 80% statistical power mainly for chondroitin sulphate (CS), and IL-1to detect a difference of 30% between the groups, with a two sided alpha level of 0.025 and β = 0.20 based on two way analysis of variance.

### Randomization

Since we tested 3 conditions (treatments), from which 2 for each horse (2 joints) we used a block design randomization. To avoid order effects for each treatment, and of the first joint used, as well as to have equal number of repetitions, we prepared cards with the possible pairs of treatments, and order of the used joint (R/L or L/R), as the tables below. The cards were kept in a box, and for each horse, the card was drawn at the time of the experiment to address concealment. Therefore, the researcher was not aware of the treatment allocation and joint that would first be used until the time for first injection (Table 2).

**Table 2:** Randomization sequence cards table.

	JOINT			JOINT	
CARD	Right (1^st^)	Left (2^nd^)	CARD	Left (1^st^)	Right (2^nd^)
1	A	B	7	A	B
2	B	C	8	B	C
3	C	A	9	C	A
4	B	A	10	B	A
5	C	B	11	C	B
6	A	C	12	A	C

A: Ozone 20μg/ml; B: Ozone 40 μg/ml; C: Oxygen (control)

### Clinical evaluation

Physical evaluation involved measurement of heart rate, respiratory rate, intestinal motility, rectal temperature, and capillary refill time as well as mucosal inspection. These measurements were performed at time 0 (control) for selection of the animals and daily thereafter until the end of the study.

Evaluation of lameness performed on day 0 (control) and daily thereafter until the end of the study, involved the following: (a) inspection for increased volume and antalgic posture; (b) palpation-for heat, pain, and alteration in consistence; (c) dynamic-evaluation for lameness, which was detected, and quantified using the Lameness Locator^®^ (Equinosis, St. Louis, MO, USA) equipment, before the treatment.

Presence of lameness was assessed objectively using equipment capable of both identifying the limb of origin and quantifying lameness. The equipment consists of a Bluetooth system, which receives the information from noninvasive wireless sensors, placed on the head (accelerometer), dorsal surface of the right thoracic limb (gyroscope), and pelvis (accelerometer). The captured movements are converted into vectors and interpreted by the program, which assigns number to the resulting vectors. For pelvic limbs, the total range of pelvic movement may not exceed ±3.

### Ultrasonography

Ultrasonography was performed on days 0, 7, 14, 21 and 28, using the ESAOTE MyLab 30 VET (Genova, Italy) ultrasound equipment, with a linear multi-frequency (7.5 to 12 MHz) electronic transducer. Ultrasonography findings were scored from 0 to 34, as described by Silva [36]: synovial fluid aspect and volume; articular capsule heterogenicity; thickening and irregularities in articular capsule insertion; intra-articular and peri-articular ligaments and tendon injuries; cartilage and subchondral changes; presence of osteophytes. The evaluators were blinded of the treatment group.

### Synovial fluid analysis

For comparative analysis of control and treated joints, SF samples (5ml) were collected before each O_2_ or O_3_ infusion on days 0, 11, 21 and 28. Arthrocentesis was performed medially to the saphenous vein, just below the medial malleolus of the tibia, in the dorsomedial face of the tarsus. The site was trichotomized and sterilized with 2% detergent chlorhexidine and 0.5% alcoholic chlorhexidine. In case of contamination with blood, SF was packed in dry tubes containing sodium heparin.

After each collection, 500μl aliquots of SF were collected for total and differential white blood cell (WBC) count and evaluation of oxidative burst by flow cytometry. The remaining SF samples were immediately centrifuged at 2000 x g at 4°C for 15 min. The supernatant was aliquoted into 2-ml tubes and frozen at -80°C for analysis of superoxide dismutase (SOD) activity and total protein, urea, glycosaminoglycan, PGE_2_, substance P, IL-1, IL-6, IL-10 and tumor necrosis factor (TNF)-α concentrations. Total WBC count was determined with a Neubauer chamber, using *in natura* aliquot; for differential WBC count, SF smears were stained in Rosenfeld stain and stored.

Total protein was quantified by the biuret method, using an automated biochemical analyzer. Urea concentrations was measured by the urease glutamate dehydrogenase method.

For analysis of glycosaminoglycans, SF samples (100 μl) were subjected to proteolysis with 4g/l maxatase (a member of the alkaline family of serine endopeptidases isolated from *Bacillus subtilis*, EC 3.4.21) in 0.05 M Tris-HCl (pH 8.0; 200 μl). After overnight incubation at 50°C, maxatase was heat inactivated (15 min; 100°C), and the debris were removed by centrifugation at 3000 x g for 10 min at room temperature. The supernatant was freeze-dried, and resuspended in 50 μl of water. Identification of SF glycosaminoglycans (hyaluronic acid [HA], and chondroitin sulphate [CS]) was accomplished by a combination of agarose gel electrophoresis (0.55%) in 0.05 M-1,3-diaminopropane-acetate buffer (pH 8.0), which allows complete separation of CS and HA and differential staining of sulfated and non-sulfated glycosaminoglycans with toluidine blue at different pH levels [37]. These compounds were quantified by densitometric analysis of electrophoresis gel slabs; all measurements were performed in duplicate (two gel slabs per sample).

The molecular weight of HA was determined by electrophoresis in 1% agarose gel in 0.04 M Tris-acetate-ethylenediaminetetraacetic acid buffer (pH 8.0; 0.02 M acetate, 0.01 M-ethylenediaminetetraacetic acid), as previously described [38]. The gels were calibrated with HA samples of known molecular weight (Select-HA^™^, Sigma-Aldrich). Two standards HA samples of known molecular weight were used on each gel slab rooster comb HA, (2 mg/ml; modal molecular weight, ~800kDa), and bovine trachea HA (1 mg/ml; modal molecular weight, ~20 kDa). Bromophenol blue was used as an indicator of distance of migration distance. After electrophoresis, the gels were stained with 0.1% toluidine blue in 0.025 M sodium acetate, (pH 5.0) for 15 min, and excess dye was removed by washing with 0.025 M sodium acetate. Migration patterns and distances were evaluated by densitometry. Migration distance was inversely proportional to the logarithm of molecular weight of HA. All measurements were performed in duplicate (two gel slabs per sample).

Synovial fluid PGE_2_, substance P, and SOD activity were quantified using a commercially available enzyme-linked immunosorbent assay kit–(Monoclonal; Cayman Chemical, Ann Arbor, MI), while IL-1, IL-6, IL-10, and TNF-α were quantified using an equine cytokine/chemokine panel (MILLIPLEX ^®^ MAP; EMD Millipore Corporation; Carrigtwahill, Co. Cork, Ireland) based on the Luminex xMAP^®^ technology. All measurements were performed in duplicate.

Oxidative burst was evaluated by flow cytometry using a FACS Calibur cytometer (BD, Franklin Lakes, NJ). Three aliquots (50 μl) of each SF sample were placed in three transparent crystal polystyrene tubes labeled as follows: 1-control, 2-basal burst, and 3-phorbol myristate acetate (PMA). Tubes 2 and 3 received 200 μl of the dichlorodihydrofluorescein diacetate reagent (final concentration, 55 μM). Additionally, the PMA tube (tube 3) received 100 μl of PMA reagent (90.9 ng/ml or ~150 nM), which acts as a chemical stimulus for oxidative burst. The final volume in all tubes was adjusted to 1.1 ml with PBS. The tubes were incubated at 37°C for 20 min. Cells were collected by centrifugation at 400 x g for 7 min at room temperature and washed with 2 ml PBS. The supernatant was discarded, and the cell pellet was resuspended in 200 μl PBS for flow cytometry. The cytometer was adjusted to collect 7,000 events, and the results were expressed as mean fluorescence intensity. All measurements were performed in duplicate.

### Statistical analysis

Data were analyzed for normality using the Kolmogorov Smirnov test. The unpaired *t*-test was used for comparison of ozone treatment groups with the control group. Comparison of post-treatment and baseline values was performed by analysis of variance, followed by Tukey’s post-hoc test. Statistical analysis was performed using the GraphPad Instat 3 (La Jolla, CA, USA). Statistical significance was set at P<0.05.

## Results

### Physical evaluation

Over the 28-day observation period, no changes were observed in intestinal motility, heart rate, respiratory rate, body temperature, capillary refill time and mucosal staining findings in any of the groups.

During evaluation of lameness, none of the horses presented with antalgic posture, sensitivity, or change in heat or consistency up on palpation. All joints presented with mild effusion throughout the study. The Lameness Locator equipment detected lameness in one horse at four time points in groups A and C. Group B exhibited at least one horse showing lameness at all time points after the beginning of treatment, with the number of lame horses being relatively high on days 11 and 14.

There were no significant differences among the groups or among different time points within the groups in term of maximum difference in pelvic height for evaluating push off lameness. However, in terms of minimal difference in pelvic height, for evaluating impact lameness, the findings of group B differed significantly from those of the other two groups on day 14 (P<0.05), the same time point when a relatively high number of horses in that group presented with lameness.

### Ultrasonography

At initial evaluation, none of the horses presented with osteochondral lesions, articular capsule enlargement, or synovial proliferation. Only three horses, one in each group, presented with increased amount of anechoic SF.

During the study period, the animals exhibited an increase in the amount of anechoic SF and synovial membrane thickness, which later decreased by day 28. Some of the horses presented with mild capsule enlargement, but with homogenous aspect. Upon IA injection, the gas was promptly absorbed, and only a small amount of gas was visualized in the dorsal aspect of the joint in the medial recess. None of the horses presented with changes in the articular line thickness, subchondral bone morphology, and or osteophyte formation during the study period.

**Fig 1:**
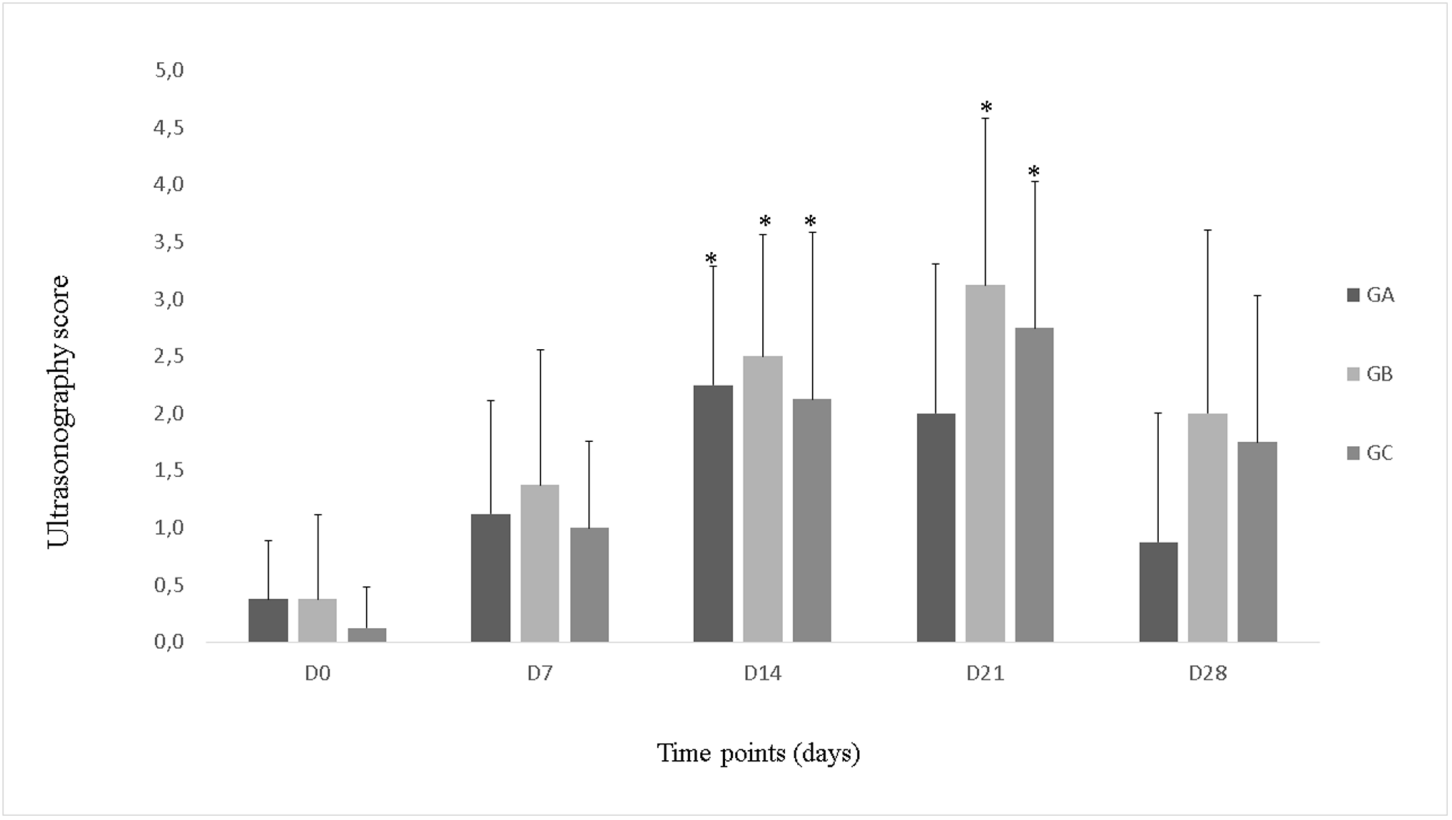
Ultrasonography score in goups A, B and C during the study period. *Statistically significant difference relative to day 0 (P<0.05).

Initial ultrasonography scores varied from 0 to 1 (out of 34) in groups A and C and 0 to 2 in group B. Owing to increase in SF volume, synovial proliferation, and articular capsule enlargement, these scores progressively increased during the treatment period until day 21. Ultrasonography scores on day 0 differed significantly from those on day 14 in all groups and from those on day 21 in groups B and C (P<0.05); the highest median ultrasonography score was 3.13. Ultrasonography scores in all groups had decreased by day 28, although the decrease was not statistically significant. There was no significant difference in ultrasonography scores among the three groups (Fig 1).

### Synovial fluid analysis

**Fig 2:**
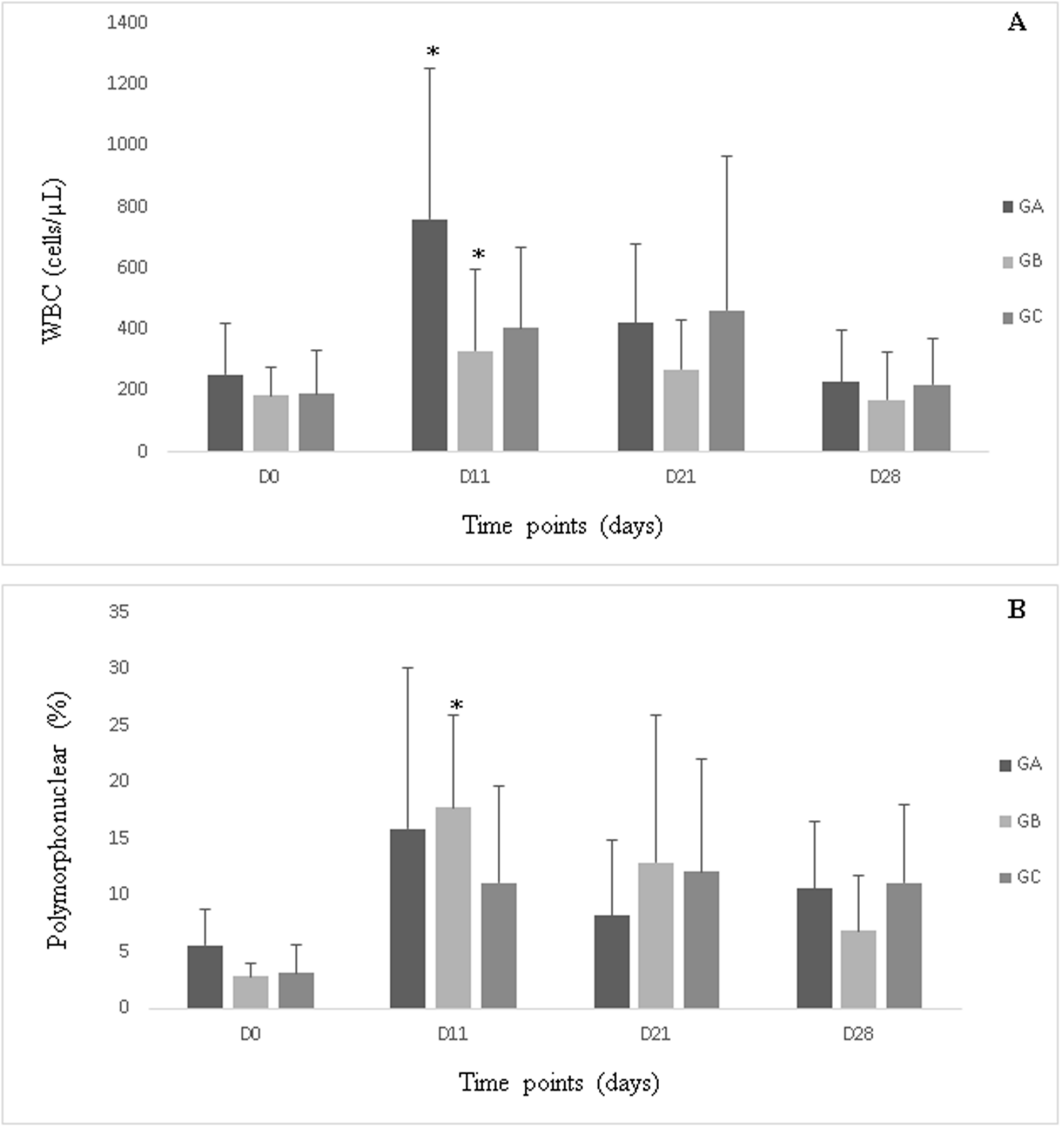
White blood cell (A, cell/μL) and polymorphonuclear cell (B, %) counts in synovial fluid in groups A, B and C during the study period. *Statistically significant difference relative to day 0 (P<0.05).

Relative to the corresponding baseline values, groups A and B exhibited increased total WBC counts on day 11 (P<0.05); however, group C did not exhibited any significant change in total WBC count, and there were no significant intergroup differences. The percentage of polymorphonuclear cells gradually increased from baseline and peaked after the fifth treatment (day 11) in group B (P<0.05) (Fig 2).

**Fig 3:**
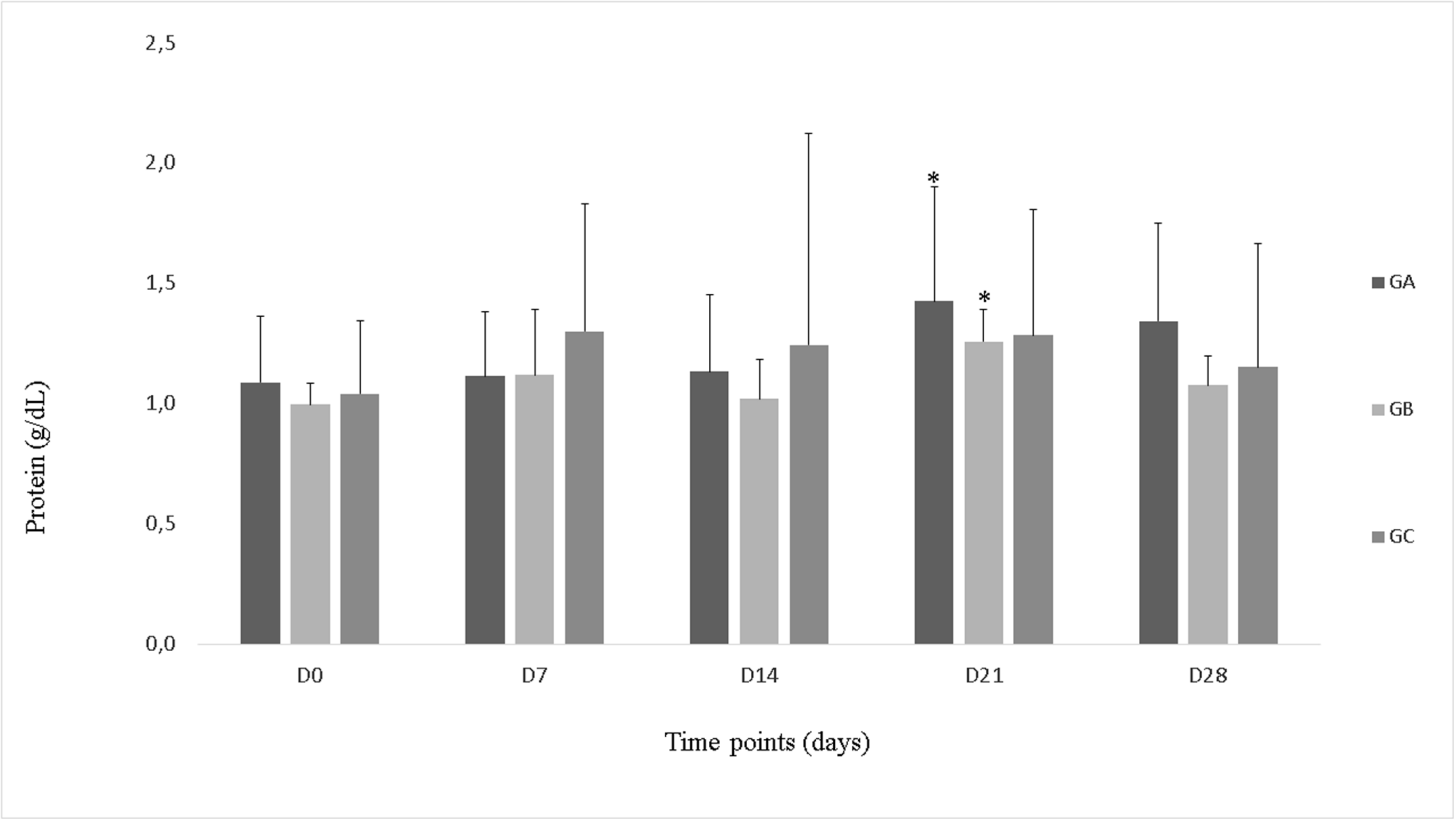
Total protein concentrations (g/dl) in synovial fluid in, groups A, B and C, during the study period. *Statistically significant difference relative to day 0 (P<0.05).

Groups A and B exhibited increased total protein concentrations on day 21 (P<0.05) in comparison to the corresponding baseline values, while group C exhibited no significant changes (Fig 3). There were no significant intergroup differences in total protein concentration.

**Fig 4:**
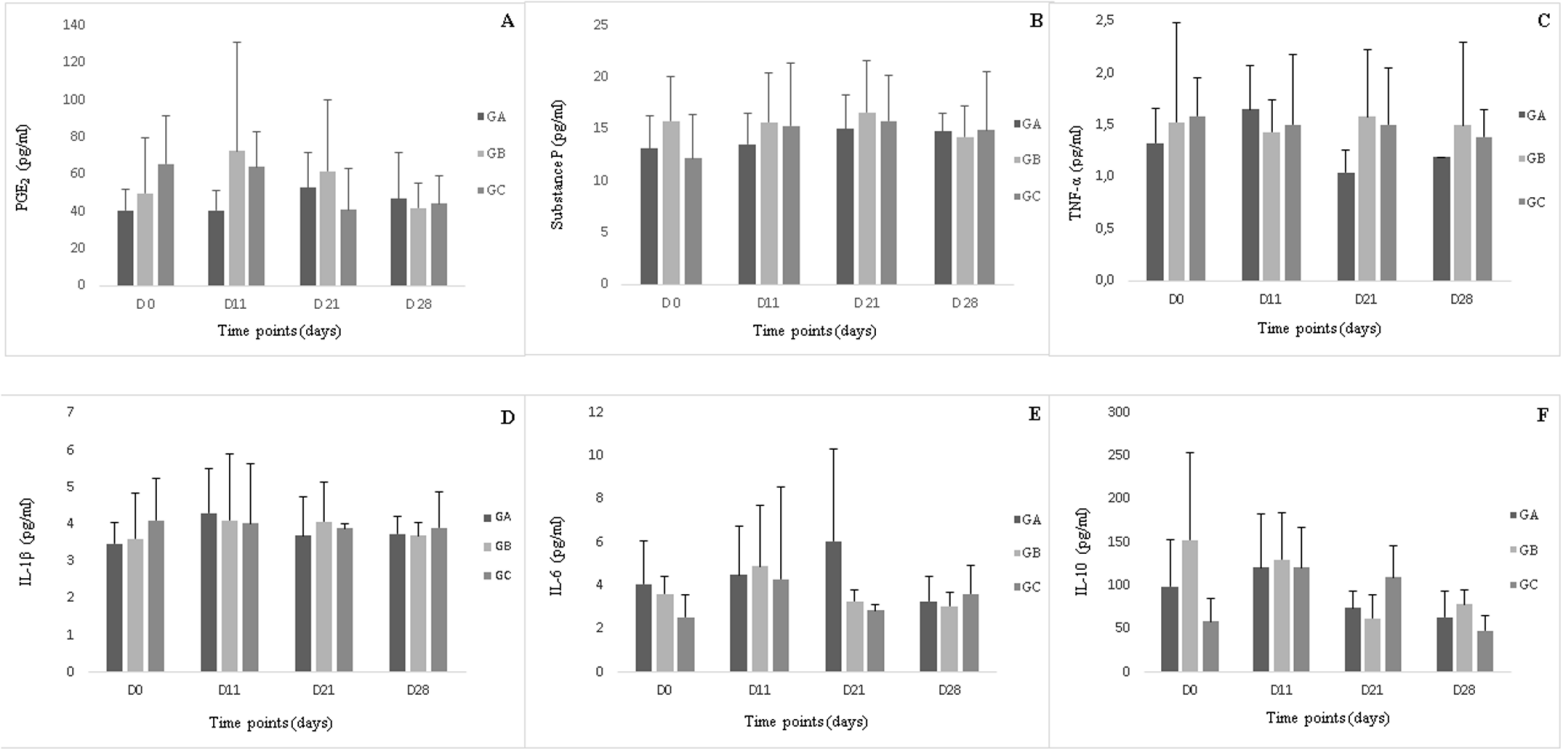
Prostaglandin E_2_ (A), substance P (B), tumoral necrosis factor-α (C), interleukin[IL]-1β (D), IL-6 (E) and IL-10 (F), concentrations (pg/ml) in synovial fluid in, groups A, B and C, during the study period.

Over the 28-day observation period, there were no significant intra- or intergroup differences in urea, PGE_2_, substance P, TNF-α, IL-1, IL-6, or IL-10 concentrations (Fig 4). Although all groups exhibited increase SOD activity, the increase was not statistically significant.

**Fig 5:**
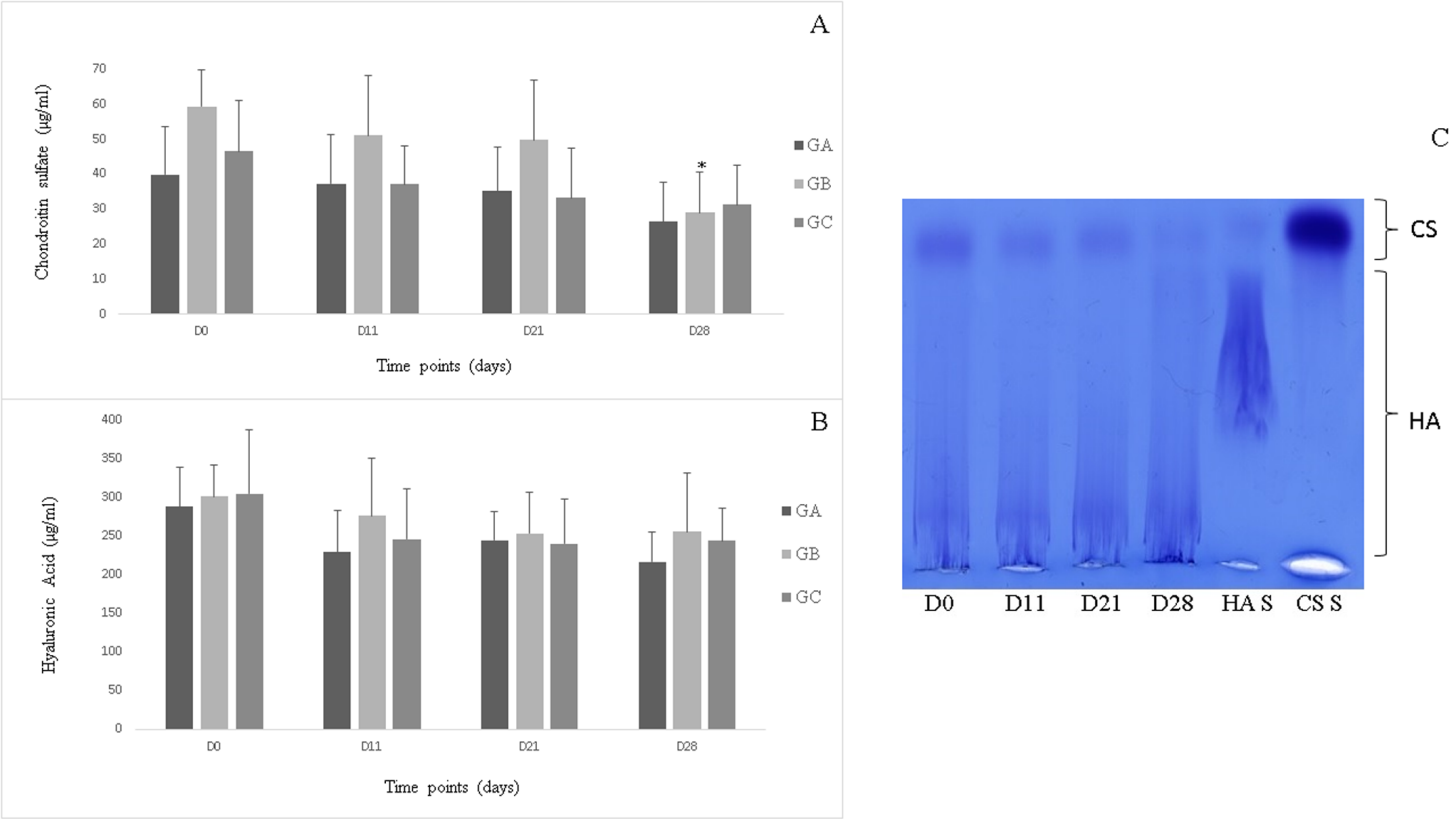
Chondroitin sulfate (A) and hyaluronic acid (B) concentrations (μg/ml) in the synovial fluid in groups A, B and C. Agarose gel electrophoresis of glycosaminoglycans isolated from equine synovial fluid samples collected during the study period (days) (C). HA = hyaluronic acid; CS = chondroitin sulphate, CS S = chondroitin sulphate standard; HA S = hyaluronic acid standard. *Statistically significant difference relative to day 0 (P<0.05).

Concerning CS concentration, only group B exhibited a decrease on day 28 (P<0.05) (Fig 5); however, there were no significant intergroup differences in CS concentration. There were no significant intra- or inter-group differences in HA concentration (Fig 5) or percentage of high-molecular weight HA.

There were no significant intra- or intergroup differences in activation index after exposure to PMA, which was used as a measure of oxidative burst.

All the measures are in the S1 Table.

## Discussion

The present study used the maximum number of treatments usually employed in routine practice to ascertain the safety of IA ozonetherapy. The results revealed, slight changes in lameness and ultrasonography findings, but no changes in the concentration of major cartilage catabolism biomarkers, cytokines and eicosanoids, which indicated the absence of significant joint inflammation. However, some of the horses, especially those in group B, presented with transient effusion and lameness, probably because of the higher dose of O_3_. This finding implies a transient inflammatory effect at this concentration of O_3_ (40μg/ml), which is consistent whit that reported in mild synovitis, that is joint effusion, caused by an increase in synovial membrane thickness and SF volume, usually accompanied by lameness [39].

Ultrasonography is a non-invasive diagnostic technique used as a control tool, because it allows monitoring of disease progression and treatment response [40], as well as visualization of articular changes—such as osteophytes formation- before X-ray radiography. Ultrasonography can also be used to evaluate soft tissues of interest to this study, such as the synovial membrane and articular capsule, as well as the quality and quantity of SF.

Changes in ultrasonography findings allowed us to conclude that repeated arthrocentesis had caused capsulitis, which was demonstrated by the puncture local thickening of the capsule, and that IA O_3_ treatments had caused mild synovitis, which had mainly resulted in the thickening of synovial membrane. Synovitis and capsulitis were responsible for the significant increase in ultrasonography scores on day 14 in all groups and on day 21 in groups B and C, which indicated that the inflammatory response in group A was brought under control by day 21. These changes in ultrasonography scores reached a maximum median score of 3.13 out of 34 during the study period, this indicates transitory mild inflammation without progression to OA, since there were no changes in cartilage line thickness, subchondral bone morphology, or osteophyte formation [41].

Analysis of SF by evaluation of total and differential WBC count and protein concentration is a routine clinical practice, which provides important information about the articular environment. Given that the normal leucocyte count—including 90% mononuclear and 10% polymorphonuclear cells—is 1000 cells/μl [42], all horses in the present study exhibited total WBC counts within normal limits at baseline. It was not observed high WBC count such as those reported by Moraes et al. [43] and Moreira et al. [44], probably because of the relatively long interval between punctures. The increased WBC counts observed on day 11 in group A and B might be attributable to the effect of O_3_ administration. In addition, group B exhibited a higher percentage of polymorphonuclear cells on day 11 in comparison to the baseline value. This change might be attributable to the inflammatory stimulus provided by the higher dose of O_3_, because neither group A nor C presented such significant changes. Moreover, on day 11,a greater number of horses in group B exhibited lameness, relative to the baseline.

The SF contains proteins at 25–35% of concentration observed in plasma [45], with a reference value of 1.8 ± 0.3 g/dl [46], and concentrations up to 2 g/dl being considered normal [47]. Increase in vascular permeability of the synovial membrane is associated with an increase in its protein concentration, which allows the transfer of high molecular-weight molecules across the barrier, thus leading to the increase in protein concentration into the SF [45]. It is known that O_3_ causes vasodilation and increased vascularization by mediating the release of nitric oxide by endothelial cells; this might have led to increased vascular permeability and, consequentily, increased protein concentrations in SF in groups A and B on day 28 [8,48,49].

Prostaglandin E_2_ is one of the more important mediators of pain and inflammatory responses [50]. Despite of Bertone, Palmer and Jones [51] consider PGE_2_ as a good to excellent predictor of OA at concentrations 22.5 pg/ml, PGE_2_ concentrations—varing from 28.5 to 65.06 pg/ml—have also been reported in other studies involving subjects with healthy joints [43,44].

The lowest median concentration of PGE_2_ observed in the present study was 38.02 pg/ml, which corresponds with the values reported by Moraes et al. [43] and Moreira et al. [44]. PGE_2_ concentrations did not vary significantly, as reported by Lamprecht and Williams [52], probably because of the relatively long interval between arthrocentesis procedures.

It was reported that at an O_3_ concentration of 20μg/ml, which was used in group A, ozonetherapy reduces PGE_2_ concentrations in SF [6,10], however, this effect was not observed in the present study, probably because we used healthy joints for evaluation.

Substance P is a neuropeptide secreted by type C nerve fiber. It exerts a direct effect on chondrocytes, resulting in increased articular pain, vasodilation, activation of macrophages, B lymphocytes, polymorphonuclear cells, platelets, and mast cells; stimulation of IL-1β, TNF-α, and IL-6 synthesis by blood cells; induction of cell proliferation; stimulation of collagenase and PGE_2_ expression by synoviocytes; increased osteoclast formation; and synovial hypertrophy [53–58]. Substance P is also related to neutrophil respiratory burst activation [59]. In the present study, no significant changes were observed in neuropeptide concentration; there was also no correlation between neuropeptide concentration and presence of lameness, which indicated that the synovitis induced by ozonetherapy was mild.

The synovial membrane produces several pro-inflammatory cytokines associated with the destruction of articular cartilage in OA, such as IL-1β and TNF-α [60]. Joints affected by OA exhibit higher IL-1β and TNF-α concentrations in SF than do health ones [61–63]. In a previous study, IL-6 concentrations were increased in diseased and non-treated carpal joints, while TNF-α concentrations neither varied according to treatment or lesion grade, nor were they associated with leukocyte or protein concentrations [64].

In another study, IL-6 was considered an excellent predictor of articular disease, and its presence always being indicative of OA [51]. In the present study, IL-6 was identified in all groups and all time points. However, because none of the other variables exhibited simultaneous changes, we cannot attribute an inflammatory character to IL-6; therefore, the presence of IL-6 in SF might be attributable to the use of a most sensitive detection method capable of detecting even low concentrations of the marker.

TNF-α and IL-1β concentrations in the present study were within the reference range suggested by Bertone; Palmer and Jones [51], and did not vary significantly. Some studies reported that ozonetherapy was capable of inhibiting IL-1β and TNF-α synthesis in animals with joint inflammation [65,66], but in this study no concentration changes were observed, probably because we used healthy joints.

In OA, increased SOD activity represents an important effect against superoxide anion and articular oxidative stress, resulting in an adaptive response through increased transformation of superoxide into hydrogen peroxide [23,67,68]. Ozonetherapy also seems to stimulate SOD activity, although this effect was not observed in the present study [9,69–73]. Activation of SOD is mediated by aldehydes, which activate nuclear transcriptional factors, and nuclear factor-erythroid-2 related factor-2, present in the cytoplasm. These, in turn, bind to the erythroid cell-derived protein associated Kelch 1 protein, interacting with nuclear antioxidant response elements and upregulating several antioxidant enzymes, including SOD [6].

Glycosaminoglycans are considered good biomarkers of articular cartilage metabolism, as demonstrated by Alwan et al. [62], Palmer, Bertone and McClain [74], McIlwraith [75], Moraes et al. [43], and Moreira et al. [44]. Fuller et al. [76] observed relatively low concentrations of HA in OA joints, although HA concentration was not correlated with cartilage damage. In the present study, there were no significant changes in HA concentrations; this is in agreement with the findings of Moraes et al. [43] and Moreira et al. [44], who observed no significant changes in HA concentrations upon joint puncture with short or long intervals.

In the present study, CS concentrations in group B decreased significantly on day 28. This is in contrast to the findings of Moraes et al. [43] and Moreira et al. [44], who reported increased CS concentrations after repeated joint puncture, as well as Fuller et al. [76] and Lamprecht and Williams [52], who did not observed any significant change. In contrast to previous studies, arthrocentesis in the present study was accompained by gas infusion, which might explain the difference in results. However, the present findings suggests that, although O_3_ treatment induced similar changes in ultrasonography and lameness findings, it did not damage the extracellular matrix, which indicates that this treatment is safe.

Joint inflammation is accompained by the degradation of high molecular-weight HA into medium- and low-molecular-weight polymers, which causes a decrease in the viscosity of SF in OA [39]. All groups in the present study exhibited a decrease in the percentage of high-molecular-weight HA; however, this tendency was lower in the O_3_ treatment groups (groups A and B) in comparison to group C, where the percentages of high-molecular-weight HA had recoverd by the end of study to levels greater than those at baseline, although the differences were not statistically significant. The total concentration of HA remained constant during the study period. Degradation of HA occurs mainly because of ROS produced by polymorphonuclear cells, primarily neutrophils, in response to respiratory burst [77]; this could justify the small decrease in percentage of high-molecular-weight HA after the start of treatment.

After articular cartilage degradation, repair mechanisms acts through diverse anti-inflammatory cytokines, including IL-10. This cytokine acts on synoviocytes, chondrocytes, and macrophages, causing a decrease in TNF-α and IL-1 concentrations [78] and inhibition of TNF-α mediated PGE_2_ secretion by synoviocytes in OA joints [79]. In the present study, it was found no correlation between increase in IL-10 concentration and decrease in the concentrations of associated inflammatory mediators. According to Bocci [6], ozonetherapy increases the concentrations of inhibitory cytokines, including IL-10. However, no significant change in IL-10 concentration was observed in the present study, probably because the evaluated joints were healthy.

The flow cytometry was used to analyze neutrophil behavior and changes in neutrophil activation index. According to Bocci [6], O_3_ treatment improves neutrophil function and faciltates neutrophil activation, thus increasing the activation index; however, no such change was observed in the present study.

## Conclusions

Consecutive treatments with IA ozonetherapy caused mild lameness, only detected by a lameness measurement system, and transient changes in ultrasonography features in horses. However, there were no significant changes in the concentrations of major biomarkers of inflammation and cartilage catabolism. It may, therefore, be concluded that IA O_3_ administration at concentrations of 20 and 40 μg/ml is safe in horses. Further studies involving medical ozone administration for treatment of different joint diseases should be undertaken to understand the benefits of ozonetherapy.

## Supporting information

S1 Table PGE_2_ (pg/ml), substance P (pg/ml), IL-1 (pg/ml), IL-6 (pg/ml), IL-10 (pg/ml), TNF-α (pg/ml), WBC (cells/μl), polimorphonuclear cells (%), heart rate (bpm), respiratory rate (mpm), temperature (°C), ultrasonography score, hyaluronic acid (μg/ml), chondroitin sulphate (μg/ml), protein (g/dl), Lameness Locator maximum difference (mm), Lameness Locator minimum difference (mm), hyaluronic acid molecular weight (%), SOD activity, activation index, urea (mg/dl). Joints and the respective group and moments, G (A, B or C) (day).
(XLSX)
